# Improvement of endothelial function by *Gunnera tinctoria* extract with antioxidant properties

**DOI:** 10.1186/s40659-020-00322-2

**Published:** 2020-11-23

**Authors:** Constanza Sabando, Maité Rodríguez-Díaz, Walther Ide, Edgar Pastene, Marcia Avello, Mario Simirgiotis, Susana Rojas, Enrique Villarroel, Tiare Silva-Grecchi, Cristian Gutiérrez, Rebeca Bouza, Bárbara Cicchelli, Marcelo González, Saddys Rodríguez-Llamazares

**Affiliations:** 1 Centro de Investigación de Polímeros Avanzados (CIPA), Avda. Collao 1202, Edificio de Laboratorios, Concepción, Chile; 2 Facultad de Medicina, Universidad Andrés Bello, República 590, Santiago, Chile; 3 Laboratorio de Farmacognosia, Departamento de Farmacia, Facultad de Farmacia, Universidad de Concepción, Concepción, Chile; 4 Laboratorio de Síntesis y Biotransformación de Productos Naturales, Universidad del Bío-Bío, Avda. Andrés Bello 720, Chillán, Chile; 5 Instituto de Farmacia, Facultad de Ciencias, Universidad Austral de Chile, Valdivia, Chile; 6 Laboratorio de Fisiología Vascular, Departamento de Fisiología, Facultad de Ciencias Biológicas, Universidad de Concepción, P.O. Box 160-C, Concepción, Chile; 7 Laboratorio de Screening de Compuestos Neuroactivos, Departamento de Fisiología, Facultad de Ciencias Biológicas, Universidad de Concepción, P.O. Box 160-C, Concepción, Chile; 8 Departamento de Microbiología, Facultad de Ciencias Biológicas, Universidad de Concepción, P.O. Box 160-C, Concepción, Chile; 9 Departamento de Física, E.U.P. Ferrol, Universidad de A Coruña, Avda. 19 de Febrero, s/n, 15405 Ferrol, Spain; 10 Laboratorio de Investigación Materno-Fetal (LIMaF), Departamento de Obstetricia y Ginecología, Facultad de Medicina, Universidad de Concepción, P.O. Box 160-C, Concepción, Chile; 11 Group of Research and Innovation in Vascular Health (GRIVAS Health), Chillán, Chile; 12 Unidad de Desarrollo Tecnológico, Universidad de Concepción, Avda. Cordillera 2634, Coronel, Chile

**Keywords:** *Gunnera tinctoria*, Methanolic extract, Endothelial function, Antioxidant activity

## Abstract

**Background:**

*Gunnera tinctoria* has been collected by Mapuche-Pewenche people for food and medicinal purposes. The high polyphenol content of methanolic extract from *G. tinctoria* leaves with chemical constituents such as ellagic acid and quercetin derivatives suggests its application to prevent endothelial dysfunction and oxidative stress. The aim of this study was to provide evidence of the protective effect of this extract on endothelial function by reducing oxidative stress induced by high d-glucose and H_2_O_2_, as well as by stimulating nitric oxide (NO) levels in human umbilical vein endothelial cells (HUVECs).

**Results:**

A methanolic extract with a high content of polyphenols (520 ± 30 mg gallic acid equivalents/g dry extract) was obtained from *G. tinctoria* leaves. Its main constituent was ellagic acid. The results of Ferric reducing antioxidant power and 2,2-diphenyl-1-picrylhydrazyl radical scavenging assays of the extract confirmed its antioxidant activity by inhibition pathway of radical species. The incubation of HUVECs with the extract decreased the apoptosis and reactive oxygen species (ROS) synthesis induced by high extracellular concentration of d-glucose or hydrogen peroxide. The extract increased endothelial NO levels and reduced vasoconstriction in human placental vessels.

**Conclusions:**

This study provides evidence about the antioxidant and endothelial protective properties of methanolic *G. tinctoria* leaf extract. The extract improves the availability of NO in HUVECs, inhibiting the production of ROS and vasoconstriction.

## Background

The collection of wild plants for medicinal purposes is an ancient tradition that has endured in the Mapuche community. *Gunnera tinctoria* (Molina) Mirb., which belongs to Gunnera genus, is widely distributed in South America and has been collected by Mapuche-Pewenche people for food and medicinal purposes. Thus, decocts of leaves, petioles or roots were prepared to assist in the treatment of dysentery [1]. Estomba et al. [2] suggested that *G. tinctoria* may have beneficial effects in curing urinary, high respiratory tract and circulatory diseases. Petioles and roots are also used against uterus pains, ulcer and liver injuries, as well as an hemostatic and anti-inflammatory remedy [3]. Zamorano et al. [4] reported that dehydrated *G. tinctoria* leaves have antioxidant capacity and antifungal activity against *Cryptococcus laurentii*. They attributed the biological activity to the high content of polyphenols in the leaves of *G. tinctoria*, where catechin and epicatechin were the main polyphenol constituents found. These flavonoids were also identified as major compounds in ethanolic extracts of *G. tinctoria* leaves [5].

In prior studies, we found that methanolic extracts of *G. tinctoria* show dermal anti-inflammatory activity for edema in mouse ear induced by 12-*O*-tetradecanoylphorbol-13 acetate [6] and arachidonic acid [7]. This pharmacological activity was attributed to the presence of polyphenols such as ellagic acid [8] and quercetin derivatives [9]. In particular, ellagic acid prevents the oxidized low-density lipoprotein (oxLDL)-induced reactive oxygen species (ROS) in human umbilical vein endothelial cells (HUVECs) [10] and inhibits NF-kappa B activity, a transcription factor involved in increasing the pro-inflammatory response [11, 12]. Based on the chemical constituents of *G. tinctoria* extracts, we suggest that its application may be beneficial in the prevention of endothelial dysfunction due to oxidative stress and inflammation.

Endothelial function is closely related to endothelium-derived nitric oxide (NO), a signaling molecule that induces relaxation in vascular smooth muscle cells, leading to reduction of vascular resistance and physiological regulation of mean arterial pressure [13, 14]. Natural nutritional products can improve endothelial function. However, relatively limited information is available about the active ingredients of food products, their bioavailability and cellular targets [15, 16].

Effective herbal drugs for treatment of endothelial dysfunction should be able to improve NO synthesis and reduce ROS synthesis and apoptosis induced by deleterious agents such as high concentration of d-glucose or hydrogen peroxide [17, 18].

HUVECs are a primary culture commonly used as an in vitro model to evaluate the effect of plant extracts on endothelial function. The antioxidative and antiglycative effects of the aqueous extract of *Psidium guajava* L. on endothelial cells exposed to glucose, glyoxal and methylglyoxal were studied using the HUVEC model [19]. The authors found that the polyphenolic compounds in the extract were potent ROS inhibitors, capable of blocking protein glycation, and had an antiapoptotic effect. Recently, it has been determined that icariin, a flavonoid glycoside isolated from the plant species *Epimedium*, can protect against high d-glucose-induced endothelial dysfunction in vitro, through attenuation of apoptosis, oxidative stress, and inflammation [18]. In general, HUVECs are considered a reliable and simple in vitro model for predicting and understanding the biological response of endothelial cells to plant extracts.

Based on the above-mentioned background, we postulate that the methanolic extract of *G. tinctoria* attenuates apoptosis and ROS generation induced by high d-glucose and H_2_O_2_, as well as stimulates NO synthesis in HUVECs, which improves endothelial function. We used two pathways to induce oxidative stress on HUVECs, exogenous supplementation of H_2_O_2_ (direct pathway) and high-D glucose [20]. In cultured HUVECs, high D-glucose increases the synthesis of superoxide through activation of nicotinamide adenine dinucleotide phosphate (NADPH) oxidase [17]. The aim of this study is to provide evidence about the protective effect of *G. tinctoria* extract on endothelial function by reducing oxidative stress induced by high d-glucose and H_2_O_2_ as well as by the stimulation of NO levels in HUVECs. The chemical constituents of methanolic *G. tinctoria* extracts are identified by high performance liquid chromatography-mass spectrometry.

## Materials and method

### Plant material

Leaves of *G. tinctoria* Mol. (Nalca) were collected in April 2012 in the Araucanía Region of Chile. Leaves were air-dried, knife grounded and sieved up to a particle size of 500 µm. The voucher material was deposited at Herbarium of Pharmacy School, Universidad Andres Bello (Collection number HPS-UNAB/14024).

### Preparation of the methanolic extract from *G. tinctoria* leaves

The methanolic extract was obtained by successive maceration of 100 g of dried plant material (average particle size < 500 µm) with hexane, dichloromethane, ethyl acetate, and methanol, at room temperature for 48 h. The solid residue/solvent ratio was 1/10 (w/v). The dispersion was continually stirred at 800 rpm. Finally, the crude extracts were concentrated under reduced pressure to dryness.

### Polyphenol content

The quantification of polyphenols was done according to the Folin–Ciocalteau method with modifications [21]. The phenolic content was expressed as mg gallic acid equivalents/g dry methanolic extract (mg GAE/g extract). The measurements were made in triplicate.

### Fractionation of *G. tinctoria* extract by pH-zone refinement using centrifugal partition chromatography (CPC)

The extract of *G. tinctoria* was fractionated using a Spot-CPC-250-B Bio-Extractor (CPC, Armen, France). The system had a four-way switching valve that allowed operation in either descending or ascending modes. The CPC unit was connected to a SPOT.PREP II system (Armen, France), with integrated UV detector and fraction collector. CPC separation was performed using the pH zone refinement method described by Ref. [22] with slight modifications. The two-phase solvent system was composed of methyl tert-butyl ether/1-butanol/acetonitrile/water with a volume ratio of 4:2:3:8. The solvent mixture was equilibrated in a separation funnel and divided in an upper and lower phase, where 10 mM trifluoroacetic acid (TFA) was added as a retainer to the upper organic phase and 10 mM NH_3_ was added as a displacer to the lower phase. The CPC rotor was first filled with 1.5 column volumes using the upper phase at 30 mL/min and 500 rpm rotation. The lower phase was pumped into the system (descending mode) at a flow rate of 12 mL/min and rotation was increased from 500 to 2000 rpm. The samples (1 g of *G. tinctoria* extract) were dissolved in 10 mL 1:1 mixture of upper (with 10 mM TFA) and lower (without NH_3_) layers and injected into the CPC system at the same time the lower phase was introduced. Elution was monitored using a scan in the range of 200–600 nm; and at 254 and 360 nm wavelengths, by collecting fractions in 32 mL tubes. Fractions with similar concentrations were combined according to on-line UV spectra and thin layer chromatography results.

### Liquid chromatography parameters and MS parameters

Fast preliminary analysis of the *G. tinctoria* extract was performed using a RP-HPLC–UV with a monolithic RP-18e column Chromolith 100–10 mm (Merck, Germany). The mobile phases employed were (A) water with 0.1% TFA and (B) acetonitrile while the gradient program was: (0.00 min, 0% B); (10.00 min, 20% B); (12.00 min, 20% B); (14.00 min, 0% B) and 2 min for column equilibration before injections. The flow rate employed was 5.00 mL/min, and the injection volume was 50 μL. Liquid chromatography-mass spectrometry was performed using an Acclaim UHPLC C18 column (Acclaim, 150 mm × 4.6 mm internal diameter, 2.5 μm, Thermo Fisher Scientific, Bremen, Germany) set at 25 ^°^C. The wavelengths used for detection were 354, 254, 280, and 330 nm, and DAD was acquired from 200 to 800 nm for full characterization of peaks. Mobile phases employed were acetonitrile (B) and 1% aqueous formic solution (A) while the gradient program was: (0.00 min, 7% B); (5.00 min, 7% B); (10.00 min, 25% B); (15.00 min, 33% B); (20.00 min, 85% B); (25.00 min, 90% B); (35.00 min, 7% B) and 15 min for column equilibration before injections. The flow rate employed was 1.00 mL/min, and the injection volume was 10 μL. Standards and the extract dissolved in methanol were maintained at 10 ^°^C during storage in the auto-sampler. The HESI II and Orbitrap spectrometer parameters were set as informed previously [23]. Peak identification was done analyzing the mass spectra, fragmentation patterns and by comparison with pure standards, accordingly.

### In vitro antioxidant activity of *G. tinctoria* extract

#### Ferric reducing antioxidant power (FRAP)

In vitro antioxidant activity of *G. tinctoria* extract was determined using the FRAP assay with modifications [24]. The FRAP reagent was prepared mixing 1020 µL of sodium acetate 300 mM at pH 3.6, 100 µL of 2,4,6-tripyridyl-s-triazine 10 mM, and 100 µL of FeCl_3_·6H_2_O 20 mM. Then, 10 µL of extract dissolution was added to the FRAP reagent. The calibration curve was prepared with FeSO_4_ solution in concentrations ranging from 15 to 35 µM and using the FRAP reagent as blank. The absorbance was measured at 593 nm in a Jenway 6405 UV–Vis spectrophometer, after 4 and 60 min of heating at 40 °C. The average of three measurements were expressed as µmol Fe^2+^/g dry methanolic extract. Quercetin was used as reference compound. Ferric reducing antioxidant power values of *G. tinctoria* extract and quercetin at 4 and 60 min were reported.

#### 2,2-diphenyl-1-picrylhydrazyl (DPPH) radical scavenging activity

The method is based on the decrease of absorbance of DPPH dissolved in methanol when mixed with an antioxidant compound. The decrease in absorbance at 515 nm is inversely proportional to the antioxidant activity. An aliquot of 100 µL of *G. tinctoria* extract were added to 3.9 mL of DPPH methanolic solution (25 mg/L). The absorbance at 515 nm was recorded at the beginning (Absorbance_onset_) and in steady state (Absorbance_end_) at room temperature, and the remnant DPPH was calculated as follows:

$$\% DPPH_{rem} = \frac{{Absorbance_{end} }}{{Absorbance_{onset} }}*100$$

The antiradical efficiency (AE) was expressed as [25]:

$$AE = \frac{1}{{EC_{50} *T_{EC50} }}$$

where EC_50_ is the concentration of antioxidant required to decrease the initial DPPH radical concentration by 50% and is obtained from a curve of remnant DPPH % versus extract concentration. T_EC50_ is defined as the time required to reach steady state at EC_50_. Gallic acid was used as reference compound. The average of three measurements were reported as L/(min × mg of extract).

### Isolation and culture of human umbilical vein endothelial cells

This investigation conforms to the principles outlined in the Declaration of Helsinki and counts with approval from the Ethics Committee of Universidad de Concepción and the Ethics and Scientific Committee of Chilean National Health System, Concepción division (CEC 19-05-3, 25-06-2019). Patient written informed consents were obtained. HUVECs were isolated by collagenase digestion (0.25 mg/mL collagenase Type I from *Clostridium histolyticum,* Invitrogen, Carlsbad, CA, USA) as previously described [26]. After isolation, cells were cultured (37 °C, 5% CO_2_) in M199 (Gibco Life Technologies, Grand Island, NY, USA) containing 5 mM d-glucose, 10% newborn calf serum, 10% fetal calf serum, 3.2 mM l-glutamine, and 100 U/mL penicillin–streptomycin (primary culture medium). Cells were used up to passage 2 and experiments were performed on cells depleted of serum (12 h) and later incubated as reported in each experiment.

### Cell viability assay

To determine the toxicity of extracts, HUVECs were seeded in 96-well plate dishes and incubated (24 h) in M199 (control) in the absence or presence of H_2_O_2_ (100 µM), and/or *G. tinctoria* extract (50, 100, 200 and 300 μg/L) or vehicle of extract (dimethyl sulfoxide, DMSO). The sample labels were chosen according to minority components added to the culture medium, thus cells incubated only with M199 were labeled as control, those incubated in presence of H_2_O_2_ as H_2_O_2_, those incubated in presence of DMSO (1% v/v) as vehicle and those incubated in presence of *G. tinctoria* extract as *G. tinctoria*. After the treatments, cytoxicity was evaluated by Alamar Blue (resazurin) assay (Thermo Fisher Scientific, Waltham, MA, USA) incubated at 10% v/v by 2 h (37 °C). Fluorescence was measured at 590 nm emission using Synergy 2 (Biotek, Winooski, VT, USA) microplate reader after 570 nm excitation. Data were expressed as a percentage of change in relative fluorescence units (RFU) compared to the average of the control.

### Live cell imaging

HUVECs were seeded in 6-well plate dishes and incubated (24 h) in M199 in the absence (labeled as control) or presence of 25 mM d-glucose (high d-glucose), H_2_O_2_ (100 µM) and/or *G. tinctoria* extract (200 μg/L) or vehicle (DMSO, 1% v/v). The sample labels were also chosen according to minority components added to the culture medium, thus those incubated in presence of 25 mM d-glucose were labeled as HG, those incubated in presence of 25 mM d-glucose and of *G. tinctoria* extract as HG+*G. tinctoria*, and those in presence of hydrogen peroxide and of *G. tinctoria* extract as H_2_O_2_+*G. tinctoria*. Imaging was performed using a Nikon Eclipse Ti-U coupled to a Nikon DS-QiMc camera (Nikon Instruments Inc., Melville, USA). Images were created by capturing phase contrast with a 40× lens. The apoptotic-like cells in each field were quantified and the rate of apoptotic-like cells was expressed in relation to the total number of cells in each field.

### Gene expression of apoptotic gene Noxa

To determine the expression of phorbol-12-myristate-13-acetate -induced protein 1 (Noxa), total RNA was isolated by chomczynski-fenol method [17] from HUVECs incubated in absence (control) or presence of high d-glucose (25 mmol/L, 24 h), H_2_O_2_ (100 µM) and/or *G. tinctoria* extract (200 µg/L) or vehicle (DMSO, 1% v/v). Non-quantitative polymerase chain reaction (PCR) was performed using Swift Max Pro thermal cycler (Esco technologies, Horsham, USA) in a reaction mixture containing 0.5 μmol/L primers, deoxynucleotide triphosphates, thermostable deoxyribonucleic acid polymerase and a reaction buffer (SapphireAmp Fast PCR Master Mix, Clontech laboratories, Mountain View, USA). The oligonucleotide primers for Noxa were gently provided by Dr. Roxana Pincheira (University of Concepción) and 28S was used as housekeeping. The amplicon images (PCR bands) in agarose gel were captured under ultraviolet (UV) light, documented and analyzed using ImageJ software (Java-based imaging processing program, National Institute of Health, USA) [17].

### Intracellular ROS levels

HUVECs were grown in 24-well plates and incubated in absence (control) or presence of high d-glucose (25 mmol/L, 24 h), H_2_O_2_ (100 µM) and/or *G. tinctoria* extract (200 μg/L) or vehicle (DMSO, 1% v/v). After treatments, cells were exposed (45 min, 37 °C) to 10 μmol/L of CM-H_2_DCFDA (Molecular Probes, Leiden, The Netherlands) in phosphate buffered saline (37 °C, pH 7.4). The fluorescence of CM-H_2_DCFDA (λexc/λem: 495/510 nm) was determined in a Synergy 2 (Biotek, Winooski, VT, USA) microplate reader [17].

### Intracellular NO levels

HUVECs were grown on microscope coverslips and incubated with M199 (labeled as Control), *G. tinctoria* extract (200 μg/L) (labeled as *G. tinctoria*), ellagic acid-enriched fraction (labeled as F3) or DMSO (labeled as vehicle, 1% v/v). To determine intracellular NO, cells were loaded (45 min, 37 °C) with 10 μM of 4-amino-5-methylamino-2′,7′-difluorofluorescein (DAF-FM) (Molecular Probes, Leiden, The Netherlands). The fluorescence was determined in fixed cells and the signal density was analyzed by ImageJ software (Java-based imaging processing program, National Institute of Health, USA) [25]. Histamine (10 μM, 5 min) was used as positive control [27].

### Wire myography in placental veins

Human placental veins of 2–3 mm external diameter were isolated, freed of adhering connective tissue, and cut into 2-mm ring segments. Two stainless steel wires were inserted through the vessel ring, one connected to a Grass FT03 force displacement transducer for isometric recording of the contractions on a Grass recorder (Model 79 D), and the other was used to adjust the resting tension vessels to 1.0 g. Vessels were bathed in modified Krebs–Ringer solution [mM: 119 NaCl, 4.6 KCl, 15 NaHCO_3_, 1.5 CaCl_2_, 1.2 MgCl_2_, 1.2 NaH_2_PO_4_, 5 d-glucose (pH 7.4, 37 °C, 95% O_2_/5% CO_2_)]. After the optimal resting tension was obtained, the tissues were allowed to equilibrate for 1 h. At the beginning and end of each experiment, the maximal contractile response to 90 mM KCl solution was recorded. Only if the contractile responses were similar in magnitude (with variation less than 10%), the data from this particular experiment were included in the analysis [28]. After maximal response to KCl, vessels were washed and equilibrated for 30–40 min. Vessels were incubated with U46619 (0.1 µM, thromboxane A2 analog) and after 5 min 200 μg/L of *G*. *tinctoria* extract were added in the experimental group. Changes in isometric tension were registered for 15 min following incubation with the extract.

### Statistical analysis

The data of FRAP assay were analyzed with a one-way analysis of variance at the 95% confidence level. Differences among the mean values of antioxidant power at 4 and 60 min were determined with Duncan’s multiple-range test.

In endothelial cell and vascular reactivity assays, the values are mean ± S.E.M., where n indicates the number of different biological samples in triplicated. Comparisons between two groups were performed by means of Student’s unpaired t-test or Mann–Whitney test for parametric or non-parametric data, respectively. The statistical software GraphPad Prism 6.0 (GraphPad Software Inc., San Diego, CA, USA) was used for data analysis; *p* < 0.05 was considered statistically significant.

## Results

### Polyphenol content and composition of *G. tinctoria* extract

**Fig. 1:**
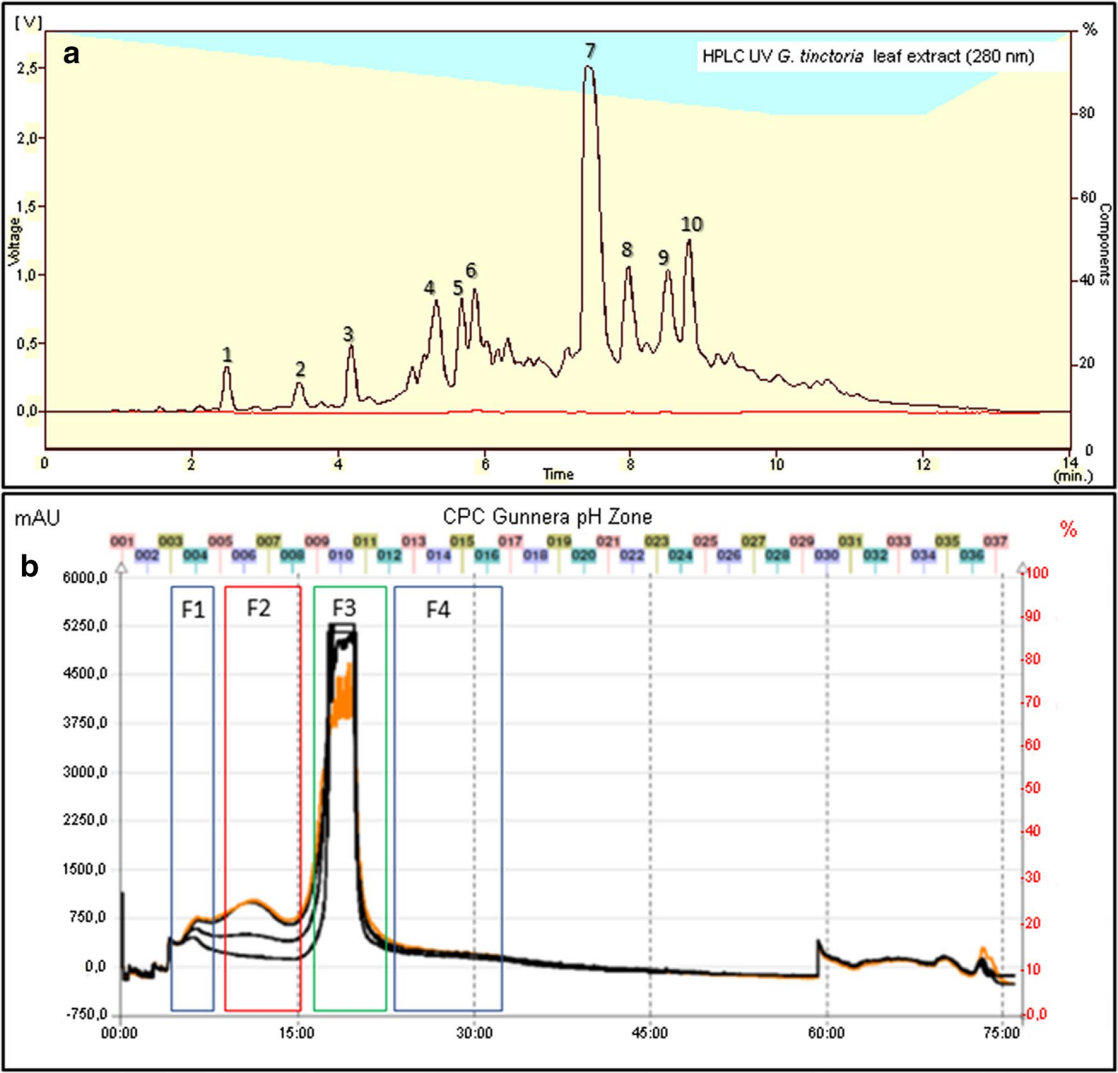
**a** HPLC profile of *G. tinctoria* extract using a monolithic RP-18 column. **b** pH-zone refinement in CPC apparatus of *G. tinctoria* extract. Peak number are the same of Table 1

The methanolic extract of *G. tinctoria* leaf showed a high polyphenol content of 520 ± 30 mg GAE/g dry extract. The HPLC–UV profile of the extract (Fig. 1a), revealed the presence of 10 compounds with a prominent peak of ellagic acid. Its concentration determined by HPLC was 166 ± 2 mg/g dry extract. After the pH zone refinement separation (Fig. 1b), four fractions (F1, F2, F3 and F4) were gathered and analyzed by LC–MS. This strategy allowed the enrichment of minor phenolic compounds of the extract for the tentative identification by High-Resolution Q-Orbitrap MS. The retention times presented in Table 1 differed from those obtained in the HPLC system with a monolithic column used for ellagic acid rapid quantification. UHPLC uses a smaller particle size to improve resolution. The identity of 9 compounds could be confirmed, being ellagic acid the main constituent. The latter could be obtained in one-step fast purification in F3 using pH-zone refinement in the Spot-CPC-250-Bio-Extractor device (Fig. 1b). Other minor phenolic derivatives can also be purified using this preparative tool (Fig. 1b and Table 1).

**Table 1:** Identification of polyphenol compounds detected in methanolic extract from *G. tinctoria* leaves by UHPLC-DAD-HR-Q-Orbitrap MS

Peak # (Fraction)	UV max	Tentative identification	Molecular formula	Retention time (min)	Measured mass (m/z)	MS^n^ ions
1 (F1)	310	p-Coumaroyl glucoside	C_15_H_17_O_8_	9.78	325.09649	–
2 (F1)	325	Digalloylglucose	C_20_H_17_O_14_	9.57	483.07800	–
3 (F1)	256–353	Rutin	C_27_H_29_O_16_	10.32	609.14611	301.03512
4 (F2)	254–354	Quercetin-3-*O*-rhamnoside	C_21_H_20_O_11_	10.63	447.09316	–
5 (F2)	325	Potentilline	C_41_H_27_O_26_	11.55	935.07501	300.99906
6 (F2)	325	Tellimagrandin	C_41_H_29_O_26_	12.23	937.08966	300.99908
7 (F3)	282, 326 (shoulder)	Ellagic acid	C_14_H_5_O_8_	12.43	300.99855	284.2; 229.3; 185.2
8 (F4)	324	Caffeic acid	C_9_H_7_O_4_	12.83	179.03441	–
9 (F4)	325	Chebulagic acid	C_41_H_29_O_27_	13.22	953.09017	300.99856
10 (F4)	255–275	Unknown	C_11_H_11_O_6_	13.53	239.0560	–

### In vitro antioxidant activity of methanolic extract of *G. tinctoria*

The ferric reducing antioxidant power assay is associated to the reduction of ferric 2,4,6-tripyridyl-s-triazine complex [Fe(III)] to ferrous 2,4,6-tripyridyl-s-triazine complex [Fe(II)]. The FRAP values for the methanolic extract and quercetin at 4 and 60 min are shown in Table 2. The FRAP value for the *G. tinctoria* extract at 60 min was higher than that at 4 min, and lower (almost half) than those obtained for the quercetin standard, a flavonoid with high antioxidant power [29].

**Table 2:** Ferric reducing antioxidant power values of *G. tinctoria* extract at 4 and 60 min

Sample	µmol of Fe^2+^/g of extract or standard	
	4 min	60 min
*G. tinctoria* extract	0.73 ± 0.01^a^	1.44 ± 0.02^a^
Quercetin	1.88 ± 0.02^b^	3.01 ± 0.14^b^

Data are shown as mean ± standard deviation (n = 3)

Values in the same column with different superscripts letters (a, b) are significantly different (p < 0.05) according to Duncan’s Multiple Range Test

The radical scavenging capacity of the extract was determined as the percentage of remaining DPPH as a function of the extract concentration. The percentage of remaining DPPH decreased with increasing extract concentration. The compounds present in methanolic extract at 5 µg/mL, minimum dose tested, reduced the DPPH by 40% (Table 3). The antiradical efficiency of the extract was 2.03 × 10^−4^ L/mg × min; being of the same order of magnitude as the reported for gallic acid (8.51 × 10^−4^), a monophenolic compound with medium antioxidant power [30].

**Table 3:** DPPH radical scavenging activity of *G. tinctoria* extract

Sample	EC_50_ (mg/L)	T_EC50_ (min)
*G. tinctoria* extract	178 ± 12	28 ± 3
Gallic acid	50.06 ± 0.01	24 ± 2

Data are shown as mean ± standard deviation (n = 3)

EC50: The antioxidant concentration that decreased by 50% the DPPH radical, estimated at steady state

TEC50: Time taking to reach steady state at EC50

### Effects of *G. tinctoria* extract on endothelial cells monolayer

**Fig. 2:**
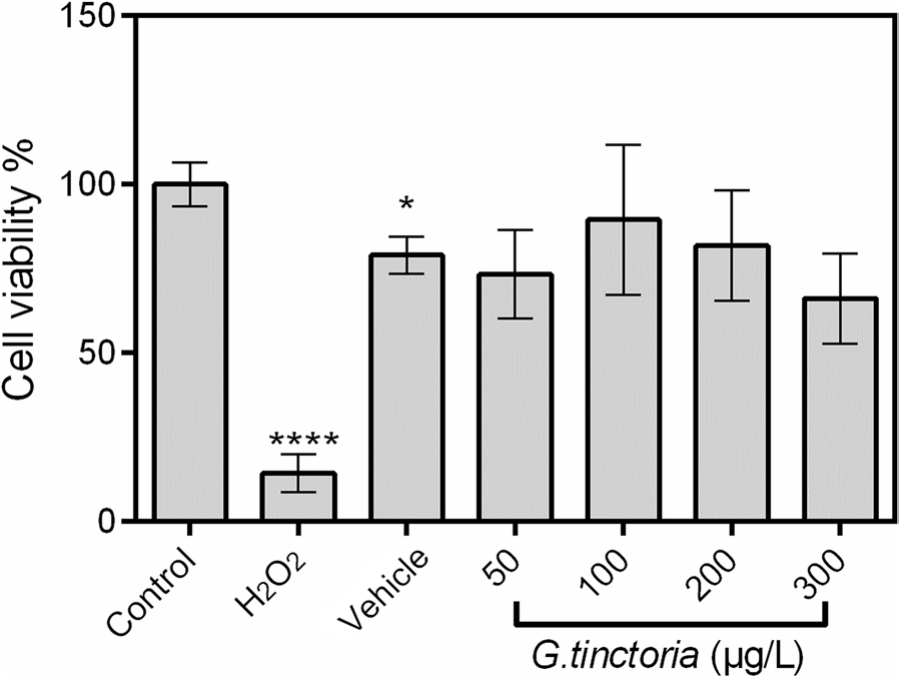
Effect of *G. tinctoria* extract on cell viability. Cell viability was determined in HUVECs incubated (24 h) with M199 in absence (control) or presence of H_2_O_2_ (100 μM), DMSO (extract vehicle) or different concentrations of *G. tinctoria* extract (50–300 μg/L). After treatments, cytotoxicity was evaluated by Alamar Blue (resazurin) assay. Cell viability was expressed as percentage (%) of mean fluorescence in control. Values are mean ± SEM, n = 4. *p < 0.05 versus control

No significant difference in cell viability was found in the incubated HUVECs (24 h) with different *G. tinctoria* extract concentrations (50, 100, 200 and 300 μg/L) compared with the control or vehicle (Fig. 2).

**Fig. 3:**
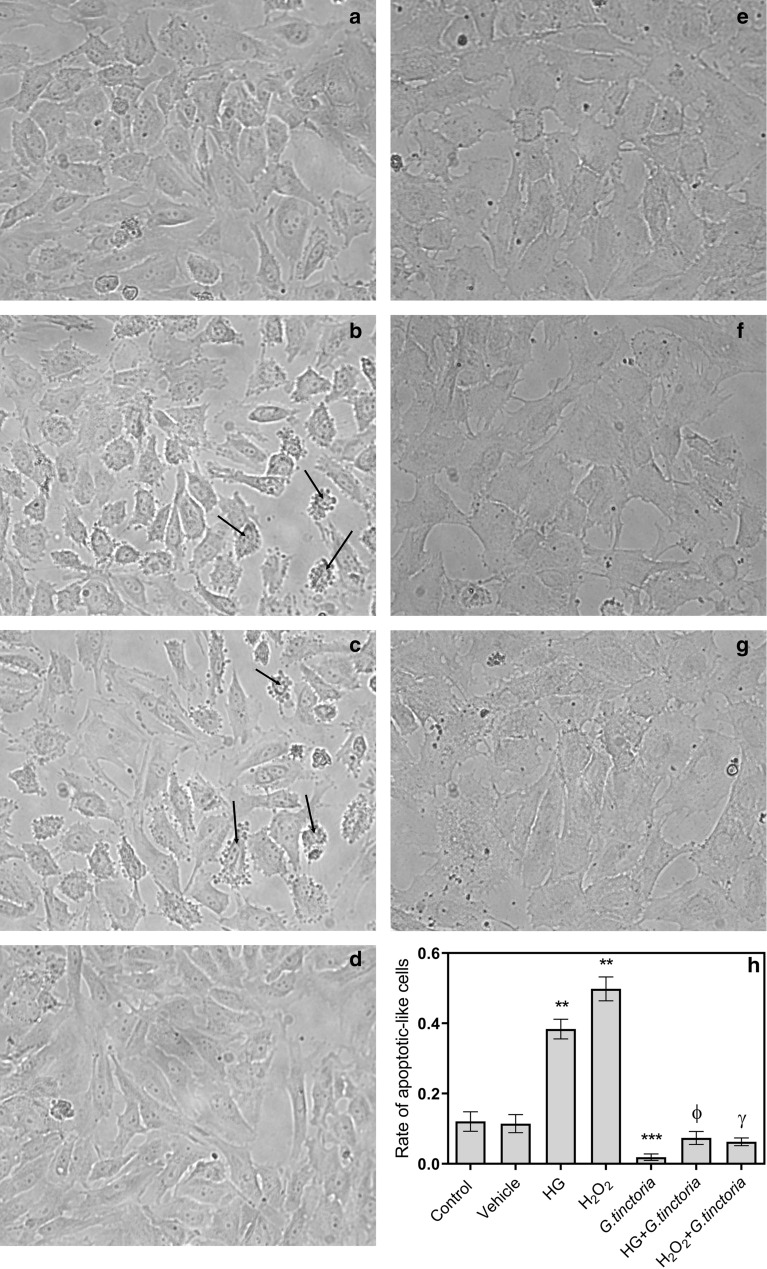
Effect of *G. tinctoria* extract on apoptotic-like cell death induced by high d-glucose and hydrogen peroxide. Representative images of light microscopy of HUVECs (×40) incubated (24 h) in M199, in absence (control) (**a**–**d**) or presence of 200 μg/L of *G. tinctoria* extract (*G. tinctoria*) (**e**–**g**). Cells were co-incubated in presence of 25 mM d-glucose (HG) (**b**, **f**), 100 µM H_2_O_2_ (**c**, **g**) or extract, vehicle (DMSO) (**d**). Black arrows in B indicate examples of apoptotic-like HUVECs. Apoptotic-like HUVECs were quantified in images from different fields (three experiments in duplicate) and result was expressed as rate of apoptotic-like cells in total cells in each field (**h**). Values are mean ± SEM, n = 3. **p < 0.01 versus control or vehicle; ***p < 0.005 versus control or vehicle; ^Ф^p < 0.05 versus high d-glucose; ^γ^p < 0.05 versus H_2_O_2_

Figure 3 shows that the incubation with *G. tinctoria* extract did not disrupt the HUVECs monolayer. Apoptotic morphology was also not evidenced in HUVECs treated with the extract. The qualitative analysis of cell morphology showed that high d-glucose and H_2_O_2_ induced cell shrinkage, membrane blebbing and formation of apoptotic bodies. These characteristics were not observed in cell cultures co-incubated with the *G. tinctoria* extract (Fig. 3e). The vehicle (DMSO) did not induce significative changes in the morphology of HUVECs (Fig. 3d). As an example, apoptotic cells are indicated by black arrows in the Fig. 3. The rate of apoptotic-like cells was increased 3.2-fold and 4.0-fold by high d-glucose and H_2_O_2_, respectively (Fig. 3b and c). The co-incubation with *G. tinctoria* extract prevented the apoptosis induced by high d-glucose and H_2_O_2_ and the rate of apoptotic-like cells returned to levels similar to the control or vehicle (Fig. 3f and g).

**Fig. 4:**
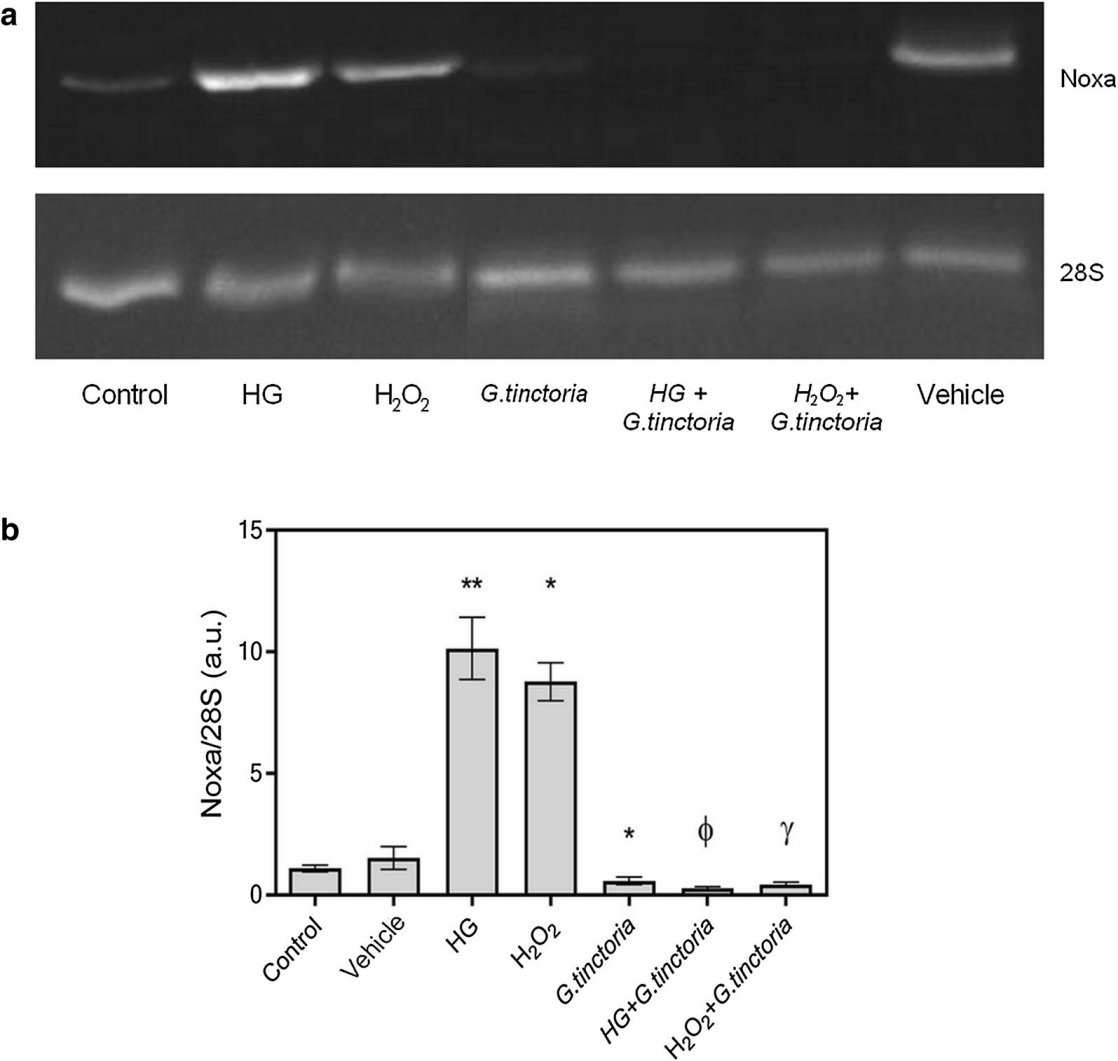
Effect of *G. tinctoria* on apoptotic gene Noxa, induced by high d-glucose and hydrogen peroxide. **a** Representative images of PCR products of Noxa and 28S obtained from HUVECs incubated with M199 and: 25 mM d-glucose (HG) or H_2_O_2_ in absence or presence of 200 μg/L of *G. tinctoria* extract (labeled as HG+*G. tinctoria* or H_2_O_2_ + *G. tinctoria*, respectively). **b** The intensity of amplicon images from three different experiments were analyzed and rate between Noxa and 28S was plotted to represent the effect of *G. tinctoria* on changes in Noxa expression in HUVECs. Values are mean ± SEM, n = 3. *p < 0.05 versus control or vehicle; **p < 0.01 versus control or vehicle; ^*Ф*^*p *< 0.05 versus high d-glucose; ^γ^p < 0.05 versus H_2_O_2_

Apoptosis was also determined by expression of Noxa. Figure 4a shows a representative image of the PCR products. In cells incubated with high d-glucose or H_2_O_2_, the Noxa expression was increased 9.3-fold and 8.0-fold compared with the control, respectively. Co-incubation with *G. tinctoria* extract suppressed the effects of high d-glucose and H_2_O_2_ on Noxa mRNA levels (Fig. 4b).

### Antioxidant effect of *G. tinctoria* extract on endothelial cells

**Fig. 5:**
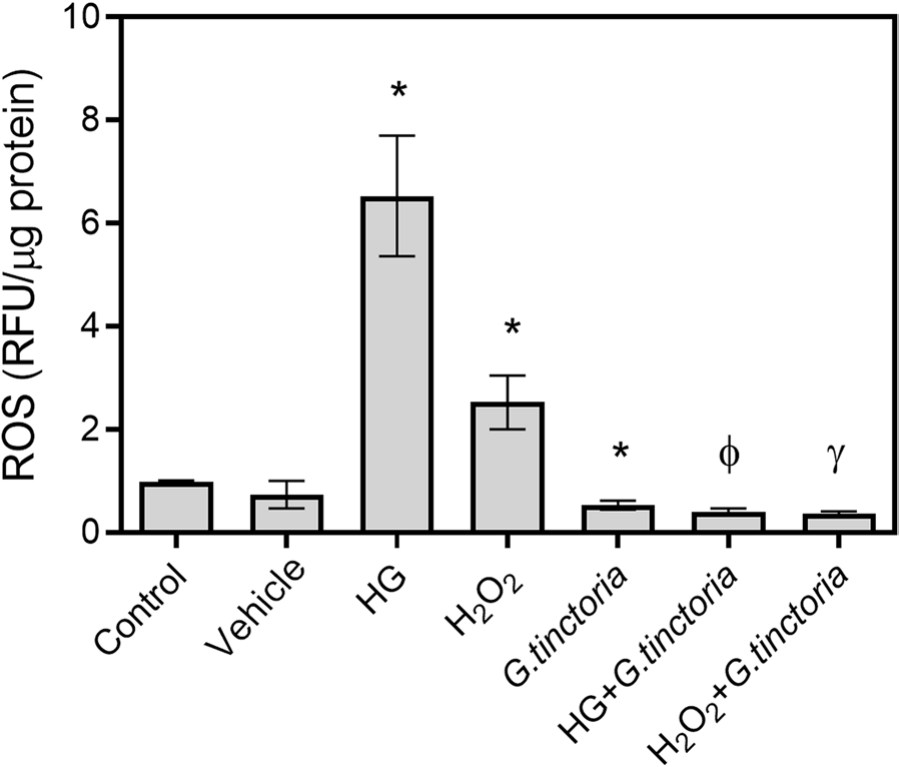
Effect of *G. tinctoria* extract on oxidative stress induced by high d-glucose and hydrogen peroxide. Reactive Oxygen Species (ROS) levels were determined by 2′,7′-dichlorofluorescein fluorescent probe in HUVECs incubated with M199 and 25 mM d-glucose (HD) or M199 and 100 μM H_2_O_2_ in absence or presence of 200 μg/L of *G. tinctoria* extract (labeled as HG + *G. tinctoria* or H_2_O_2_+*G. tinctoria*, respectively). Changes in ROS levels are shown as rate of control. Values are mean ± SEM, n = 3. *p < 0.05 versus vehicle, ^Ф^*p *< 0.05 versus high d-glucose; ^γ^*p *< 0.05 versus H_2_O_2_

The *G. tinctoria* extract decreased the ROS content by 50% compared to control. The equivalent concentration (v/v) of vehicle did not influence the ROS production. High d-glucose or H_2_O_2_ increased the ROS level compared to control 5-fold and 1.9-fold, respectively. The co-incubation with *G. tinctoria* extract blocked the ROS production induced by high d-glucose and H_2_O_2_ (Fig. 5).

### Effect of *G. tinctoria* extract on NO levels

**Fig. 6:**
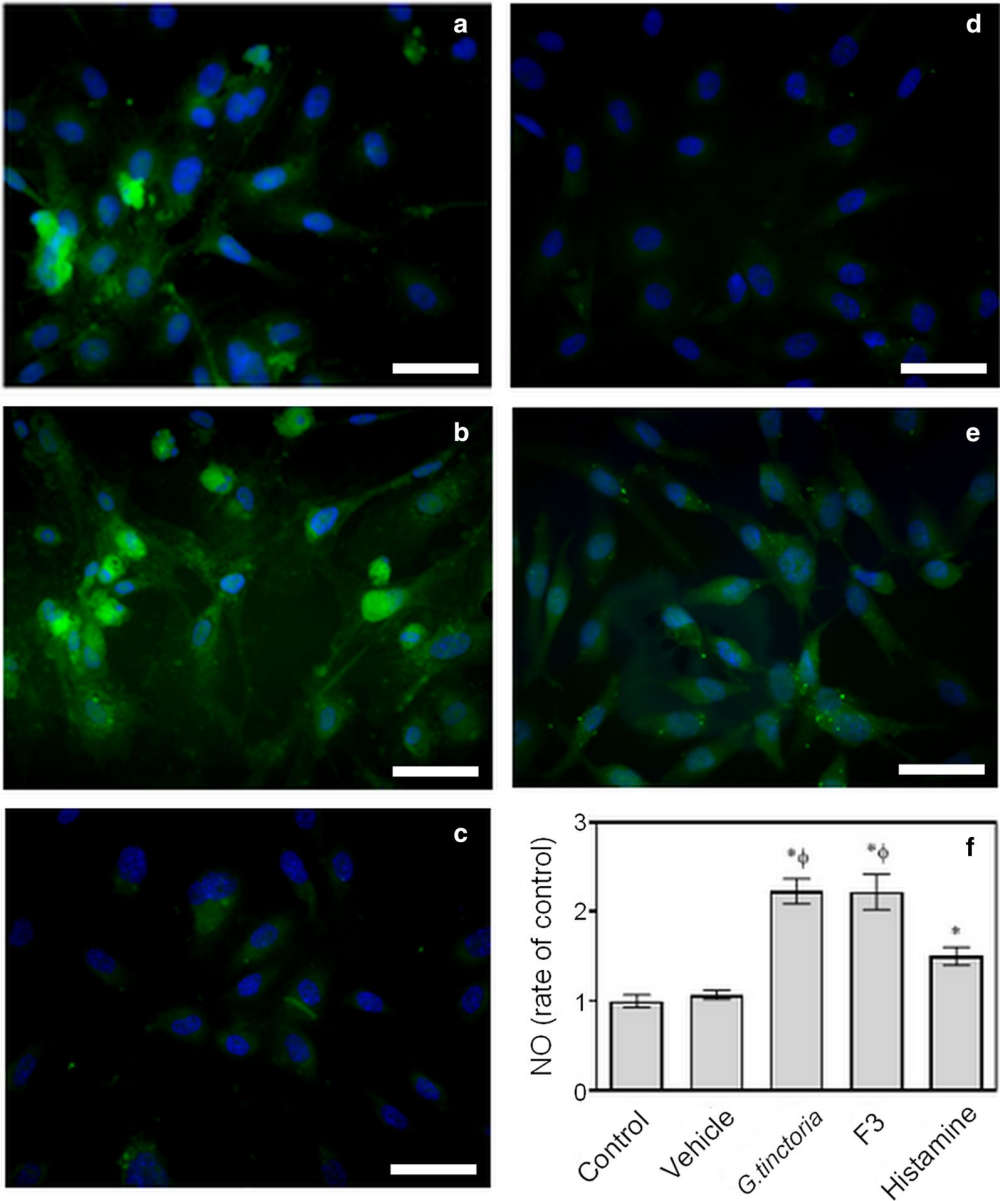
Effect of F3 fraction of *G. tinctoria* extract and crude extract on nitric oxide levels. Nitric oxide (NO) levels were determined with DAF through fluorescence microscopy in HUVECs incubated (37 °C, 24 h) with M199 in absence (Control) (**c**) or presence of 200 μg/L of *G. tinctoria* extract (labeled as *G. tinctoria*) (**a**), 200 μg/L of ellagic acid-enriched fraction (labeled as F3) (**b**) or vehicle (**d**). Positive control were cells incubated (37 °C, 5 min) with 10 μM histamine (labeled histamine) (**e**). In images, green fluorescence corresponds to DAF probe and blue fluorescence corresponds to 4′,6-diamidino-2-phenylindole, for nuclei staining. The density of DAF fluorescence per cell was quantify using ImageJ software and was expressed as rate of control (**f**). Values were expressed as mean ± SE, n = 3. *p < 0.05 versus Control; ^Ф^*p *< 0.05 versus vehicle. The scale bar represents 50 μm

Enhanced DAF fluorescence in cytoplasm of cells was observed in HUVECs incubated with the *G. tinctoria* extract or an ellagic acid-enriched fraction (F3). Fluorescence in cells incubated with *G. tinctoria *extract was more intense than fluorescence in cells incubated with histamine, but fluorescence distribution was similar in both (Fig. 6). Quantification of fluorescence intensity revealed that the *G. tinctoria* extract and the ellagic acid-enriched fraction (F3) increased NO levels 2.2-fold in HUVECs, compared with the control or vehicle.

### Effect of *G. tinctoria* extract on contractile response of human placental veins

**Fig. 7:**
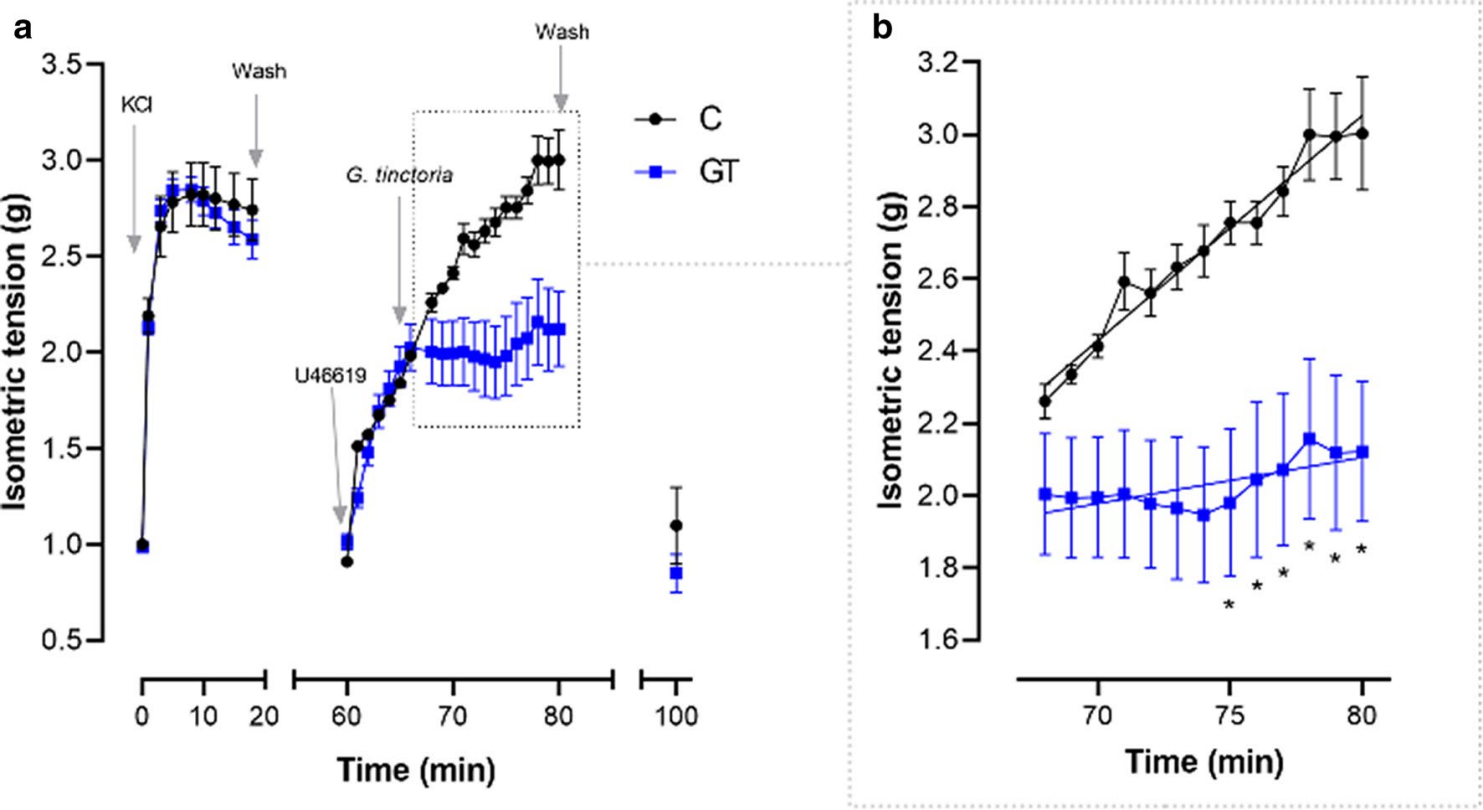
Blockade of contractile response to U46619 induced by *G. tinctoria* extract. Isometric tension of rings of human chorionic vein vessels incubated (37 °C, 95% O_2_/5% CO_2_) in absence (black circles, labeled C) or presence of 200 μg/L *G. tinctoria* extract (blue squares, labeled as GT) and previously exposed to 0.1 μM U46619 (thromboxane A2 analog). **a** The changes in isometric tension are shown after adjustment and equilibration of vessels to 1.0 g. The addition of KCl solution (90 mM), physiological buffer (Wash), U46619 and *G. tinctoria* are indicated with arrows. **b** The isometric changes after incubation with *G. tinctoria* (section highlighted in **a**) is shown in detail. U46619 was not removed. The slopes for U46619 constriction, calculated from linear regression, are 0.062 ± 0.002 (g/min) and 0.013 ± 0.002 (g/min) for control and *G. tinctoria*, respectively. Values are mean ± SEM, n = 5. *p < 0.05 versus control (C)

The increase of constriction induced by thromboxane A2 analog, U46619, was significantly reduced by 200 μg/L *G. tinctoria* extract (Fig. 7).

In summary, the presence of apoptotic endothelial cells induced by high levels of d-glucose or H_2_O_2_ was significantly reduced by co-incubation of HUVECs with *G. tinctoria* extract, which is confirmed by the blocking of Noxa expression. The ability of the extract to reduce high d-glucose- and H_2_O_2_-induced ROS is congruent with the reduction of U46619- induced vasoconstriction and the stimulation of NO synthesis.

## Discussion

*Gunnera tinctoria* is commonly harvested for its edible petiole. Their leaves and roots are also used for food and medicinal purposes. The beneficial effects of its leaves are attributed to their high polyphenol content. We found gallic acid equivalents of 520 mg/g extract, where almost 32% of the leaf extract was ellagic acid. The reported polyphenol content of a methanolic extract of *Gunnera perpensa,* another species of the genus *Gunnera*, was much lower (248.45 mg GAE/g extract) compared with the values found by us for the *G. tinctoria* leaf extract [31].

Ellagic acid, the main constituent of the *G. tinctoria* extract, with four phenolic and two lactone groups, can act as hydrogen bond donor and acceptor which makes it a powerful antioxidant [32]. Several phenolic compounds in extracts of *Gunnera* genus have been identified. Fifteen flavonoids (glycosides of quercetin, kaempferol and isorhamnetin) have been isolated from leaves of endemic *Gunnera* species grown in Juan Fernández island, Chile: *G. bracteata*, *G. masafuerae* and *G. peltata* [33]. The authors found that glycosides of isorhamnetin are flavonoids characteristic of these species of *Gunnera* genus. *G. tinctoria* is considered a likely progenitor for endemic species of Juan Fernandez, according to morphological and flavonoid data. In other species of *Gunnera* genus such as *G. densiflora,* pelargonidin 3,5-diglucoside is the main polyphenol found in leaf extracts [34]. Gallic acid is also identified as a predominant phenol in aqueous and methanolic extracts of leaves of Brazilian *G. Manicata* [35]. Ellagic acid and its derivatives have been identified only in *G. perpensa*. The glucoside of ellagic acid, trimethyl ether, 4-*O*-β-d-glucopyranosyl-3,3′,4′-tri-O-methyl-ellagic acid, was isolated from aqueous extract of *G. perpensa roots* [36]. Mayori et al. [37] reported other phenolic lactones isolated from methanolic extracts of *G. perpensa* root, such as ellagic acid and 3,3′,4-tri-*O*-methyl ellagic acid. Mammo et al. [38] also described the presence of another glucoside of ellagic acid isolated from *G. perpensa*, punicalagin. However, ellagic acid or its derivatives are not reported as majority compounds in *G. perpensa* species. Note that ellagic acid is considered a strong antioxidant, capable of scavenging free radicals such as hydroxyl, peroxyl, NO_2_ and peroxynitrite [39].

FRAP and DPPH results for methanolic extracts confirm their antioxidant activity by inhibition pathway of radical species. The methanolic extracts of roots, rhizomes and bulbs of *G. perpensa* also reduce DPPH radicals [32, 40]. The antioxidant response of these extracts is related to the content and chemical structure of polyphenols, and their interactions with other antioxidants. In general, plant extracts with free ellagic acid and ellagitannins have a higher antioxidant activity [8]. The strong activity of *Punica granatum* L. juice is attributed to its polyphenols, including punicalagin and ellagic acid [41]. However, *P. granatum* juice and juice extract showed superior antioxidant activity compared to its purified polyphenols. Therefore, the antioxidant effect is not only attributed to polyphenolic compounds, but also to terpenoids and steroids [42, 43].

In the case of the *G. tinctoria* extract, we found no difference in NO levels between the application of the crude extract or the enriched fraction in ellagic acid (F3). It has been found that ellagic acid is a compound with proven properties in the prevention of endothelial dysfunction associated with atherosclerosis and inflammation. Thus, Lee et al. [10] showed that the pre-treatment of HUVECs with ellagic acid (5–20 µM) followed by exposure to oxLDL inhibits the expression of lectin-like receptor for oxLDL (LOX-1). The decrease of LOX-1 induced by ellagic acid attenuates the NADPH oxidase abundance in the endothelial cell membrane, which results in the decrease of intracellular ROS formation. The incubation with ellagic acid also restores the endothelial nitric oxide synthase expression decreased by oxLDL. Mele et al. [44] found that the treatment of HUVECs with ellagic acid (5–10 µM, 6 h) decreases the monocyte adhesion, the soluble vascular cell adhesion molecule-1 and interleukin-6 secretion, induced by tumor necrosis factor-α.

*G. tinctoria* extract delays ROS production in endothelial cells induced by high d-glucose (25 mM), a stimulus that increases the synthesis of superoxide through activation of NADPH oxidase in HUVECs [17]. Considering that incubation with ellagic acid (20 µM) prevents the increase of ROS induced by high d-glucose (30 mM) in human aortic endothelial cells [45], a potential mechanism for the antioxidant effect of *G. tinctoria* extracts could be related to the regulation of expression and activity of NADPH oxidase by ellagic acid. NADPH oxidase is the main source of ROS in the endothelium [17], so the down-regulation of its expression in hyperglycemia is a key mechanism for improving endothelial function. The incubation with ellagic acid also induces endothelium-dependent vasodilation in rat aortic rings [46], which could be related to the reduction in U46619-induced constriction by the *G. tinctoria* extract. Previously, it has been determined that endothelial-protective stimuli, like insulin, decreases the constriction induced by U46619 in placental veins, by a mechanism dependent on endothelium-derived NO [47]. Further studies are required to determine whether the effects of the *G. tinctoria* extract on NO synthesis or vascular reactivity depend on NOS activity. For example, by co-incubation of the extract with N(ω)-nitro-l-arginine methyl ester and endothelium denudation in vascular reactivity assays.

The *G. tinctoria* extract showed an anti-apoptotic effect associated with regulation of Noxa expression. In addition, it has been determined, that homocysteine, a molecule related to endothelial dysfunction and cardiovascular disease [48], increases the Noxa expression in HUVECs. This effect is blocked by the NO donor S-nitroso-N-acetylpenicillamine [49]. This evidence suggests that the mechanism involved in the regulation of Noxa expression and apoptosis by the extract could be related to the increase of induced NO. Thus, the mechanism for vascular actions of the extract could involve the activity of ellagic acid on a signaling pathway that decreases the NADPH oxidase-derived ROS and increases the activity of endothelial NOS, improving the endothelium-derived NO availability.

## Conclusion

This study provides scientific evidence on the ethnomedicinal use of *G. tinctoria* leaves in Chilean folk medicine. The methanolic *G. tinctoria* extract has a positive effect on endothelial function in pathophysiological conditions of high d-glucose and H_2_O_2_: it increases NO synthesis, reduces ROS levels and inhibits the apoptosis in HUVECs. The improved endothelial function and antioxidant activity of the extract suggests its potential application in the formulation of phytomedicines for the treatment of a wide range of diseases.

## Data Availability

The datasets generated in the current study are available from the corresponding authors on request.

## Abbreviations

AE: Antiradical efficiency
ApoE-/-: Atherosclerosis-prone apolipoprotein E-deficient
CM-H_2_DCFDA: General oxidative stress indicator
CPC: Centrifugal partition chromatography
DAD: Diode-array detector
DAF: Diaminofluorescein
DMSO: Dimethyl sulfoxide
DPPH: 2,2-Diphenyl-1-picrylhydrazyl
EC_50_: Median effective concentration
ERK1/2: Extracellular signal-regulated protein kinases 1 and 2
FRAP: Ferric reducing antioxidant power
F3: Ellagic acid-enriched fraction
GAE: Gallic acid equivalents
HG: High d-glucose
HO-1: Heme oxygenase 1
HPLC: High-performance liquid chromatography
HR: High resolution
HUVECs: Human umbilical vein endothelial cells
H_2_O_2_: Hydrogen peroxide
LC-MS: Liquid chromatography–mass spectrometry
LOX-1: Lectin-like oxidized LDL receptor-1
M199: Medium 199
NO: Nitric oxide radical
NADPH: Nicotinamide adenine dinucleotide phosphate
NOS: Nitric oxide synthase
NOX4: Nicotinamide adenine dinucleotide phosphate oxidase 4
oxLDL: Oxidized low-density lipoprotein
PCR: Polymerase chain reaction
Q-Orbitrap MS: Quadrupole orbitrap mass spectrometry
RFU: Relative fluorescence units
RNA: Ribonucleic acid
ROS: Reactive oxygen species
RP: Reverse phase
SEM: Standard error of the mean
TEC_50_: Time needed to reach equilibrium at the EC_50_
TFA: Trifluoroacetic acid
UHPLC: Ultra-high-performance liquid chromatography
UV: Ultraviolet
U46619: Thromboxane A2 analog
